# Dental Hygiene

**Published:** 1873-11

**Authors:** W. H. Jackson

**Affiliations:** Ann Arbor, Mich.


### DENTAL HYGIENE.

BY DR. W. H. JACKSON, ANN ARBOR, MICH.,

*Read before the Michigan State Dental Association.*

Hygiene is the art of preserving health; that is, of obtaining the most perfect action of body and mind during as long a period as is consistent with the laws of life. In other words, it aims at rendering growth more perfect, decay less rapid, life more vigorous, death more remote.

Dental hygiene is the art of preserving the teeth; yet it is so closely connected with general hygiene, that it seems almost impossible to separate the one from the other; in fact, I am unable to draw the dividing line, for that which gives the most perfect health should also preserve the teeth to the greatest age, as decay in the teeth is nothing but disease of those organs, or the result of disease in associate organs, or both.

First, teeth may decay owing to a diseased formation of their tissues during growth, or from a starved condition of the system in reference to the materials which go to build up the osseous structure.

Second, decay (dental) may arise from vitiated secretions of the mucous membrane of the oral cavities and of the salivary glands, regurgitations from a disordered stomach, want of cleanliness, accidents, etc.

As is readily seen, this subject carries us back beyond infancy, even before the formation of the cell which contains the germ that is to be quickened into life. Some may think that this is going too far, yet who will say that the child does not resemble its parents? If they are weak and sickly, as a general rule the child will be sickly; if their teeth are badly decayed, the child will almost universally loose its teeth in the same manner. This resemblance may be traced back sometimes to the father, and sometimes to the mother, yet I believe it is more frequently traceable to the latter.

I have no doubt that these hereditary tendencies, many times, may be modified, if not entirely arrested; still there may be a hereditary physiological condition of the organs which build up the dental tissues which it is impossible to modify; such as the shape of the teeth, the absence of certain teeth in the mouths of some families, and the general structure of the tooth substance.

In accordance with the belief of most physiologists, the thoughts and emotions of the mother's mind influence the future character of the child. In like manner, I believe that the future condition of the child is influenced by the materials which form the pabulum that feeds the child during intra-uterine life.

We are too apt to attribute the poorly ossified teeth of the child to a hereditary predisposition to decay, when in fact it is not properly a pathological condition of the organ that forms them, but it is the absence of the necessary materials with which to build a perfect structure.

The crowns of the temporary teeth are formed during intra-uterine life. During that period the mother is called upon for a greater supply of the inorganic materials which are required for the formation of the osseous system of the child. How is the mother to comply with the demand? Upon examination we find that a sufficient quantity is not contained in the food she eats, or the water she drinks. What is the result? The mother is deprived of a portion of that which should have gone to repair the changes taking place in her own osseous system, the effects of which are so often seen in the rapid decay of her teeth during gestation and lactation. I have seen several cases where, in the short period of nine months, good substantial sets of teeth, which ought to have lasted during their natural life, pass beyond all hope of recovery from no other apparent cause.

The effect upon the child is equally bad, for the supply is limited until the crowns of the temporary teeth are formed, and the organs which deposit the osseous substance (and I might add, the nerves which govern these organs) have formed the habit of doing their work poorly, or perhaps ceased to act entirely on account of continued forced idleness. As an illustration of this, you will generally find in strabismus, that during infancy the eyes have nearly equal power. By use, one eye becomes strong, the other becomes weak from want of use; and after a time the weak eye nearly, and sometimes quite, loses its power of sight. A person who becomes by accident or disease absolutely deaf, is very apt to lose his speech gradually, until he is unable to articulate at all.

What is necessary to be done, then, is to supply the mother with an abundance of those materials which go to form the teeth. If they are not to be found in the different articles of food the patient eats, supply them artificially in the form of hypo-phosphite, lacto-phosphate, or common phosphate of lime. If these are not to be obtained, aqua calcis may be used with care. During childhood the same should be given whenever there is any tendency toward caries or imperfect development. In cases of low vitality, it may be necessary to give tonics and stimulants to arouse the system to increased action.

I do not regard the phosphate of lime so much a medicine as a food which the body requires for the building up and preservation of the teeth and bones. Deprive them of it, they will starve just the same as the soft tissues would, should the inorganic food be in excess of the organic ; and one is just as essential to perfect health as the other.

Hence it is necessary that the parents have perfect, healthy teeth, in order that the child may inherit the same tendency toward perfect dentition. If the parents have poor teeth, it is the duty of the dentist to give such treatment as will place the parents' systems in the most favorable condition possible, that the child may not inherit the curse already upon the parents. In my own practice, I have often seen, during gestation, the mother's teeth decaying rapidly, showing a white line along the margin of the gums and extreme sensitiveness at the necks of the teeth. These symptoms have nearly always disappeared by the administration of the phosphates, the white line disappearing and the teeth becoming hard. In such cases the mother should continue to take the phosphates during the full period of gestation, also during lactation ; and whenever, during adolescence or adultage, symptoms of decay appear, the same treatment is indicated. To be sure, there are times when the system is generally diseased, also when the associate organs are diseased; that the secretions cannot be corrected without other treatment; and it should be one of the qualifications of the dentist, to properly diagnose and treat these pathological conditions which come within his jurisdiction; for without such knowledge, he is unable to preserve the teeth, because of the improper surroundings. We would uot think of trying to live in a room filled with carbonic acid, or one in which a great proportion of the atmosphere was carbonic acid, because we have found that it destroys life; yet we allow our patients to come to us for advice, and we allow them to go away without giving them anything to correct the secretions of their mouths, while their teeth are surrounded by fluids which are as detrimental to their teeth as carbonic acid is to the respiratory organs.

But for me to write an article covering all that comes under this head, would impose on your good nature too much, were I capable, and had the will to do so.

You have all read of the necessity of cleanliness, such as removing all calculi that may adhere to the teeth, also removing whatever food may become lodged between the teeth, brushing thoroughly, and removing superficial caries; and I have no doubt that most of you would proceed, in case you should find a cavity, to fill the same; so without trespassing any farther upon your time, I will close this already too long article.

